# Enhanced differentiation of human pluripotent stem cells into cardiomyocytes by bacteria-mediated transcription factors delivery

**DOI:** 10.1371/journal.pone.0194895

**Published:** 2018-03-26

**Authors:** Yongxin Jin, Ying Liu, Zhenpeng Li, Katherine Santostefano, Jing Shi, Xinwen Zhang, Donghai Wu, Zhihui Cheng, Weihui Wu, Naohiro Terada, Shouguang Jin, Fang Bai

**Affiliations:** 1 State Key Laboratory of Medicinal Chemical Biology, Key Laboratory of Molecular Microbiology and Technology of the Ministry of Education, Department of Microbiology, College of Life Sciences, Nankai University, Tianjin, China; 2 Department of Pathology College of Medicine, University of Florida, Gainesville, Florida, United States of America; 3 Key Laboratory of Regenerative Biology, Guangzhou Institute of Biomedicine and Health, Chinese Academy of Sciences, Guangzhou, China; 4 Department of Molecular Genetics and Microbiology, University of Florida, Gainesville, Florida, United States of America; University of North Dakota, UNITED STATES

## Abstract

Virus-mediated expression of defined transcription factor (TF) genes can effectively induce cellular reprogramming. However, sustained expression of the TFs often hinders pluripotent stem cell (PSC) differentiation into specific cell types, as each TF exerts its effect on PSCs for a defined period of time during differentiation. Here, we applied a bacterial type III secretion system (T3SS)-based protein delivery tool to directly translocate TFs in the form of protein into human PSCs. This transient protein delivery technique showed high delivery efficiency for hPSCs, and it avoids potential genetic alterations caused by the introduction of transgenes. In an established cardiomyocyte *de novo* differentiation procedure, five transcriptional factors, namely GATA4, MEF2C, TBX5, ESRRG and MESP1 (abbreviated as GMTEM), were translocated at various time points. By detecting the expression of cardiac marker genes (*Nkx2*.*5* and *cTnT*), we found that GMTEM proteins delivered on mesoderm stage of the cardiomyocytes lineage differentiation significantly enhanced both the human ESC and iPSC differentiation into cardiomyocytes, while earlier or later delivery diminished the enhancing effect. Furthermore, all of the five factors were required to enhance the cardiac differentiation. This work provides a virus-free strategy of transient transcription factors delivery for directing human stem cell fate without jeopardizing genome integrity, thus safe for biomedical applications.

## Introduction

Cardiovascular disease is a leading cause of human death worldwide [1, 2]. The limited capability of heart tissue to regenerate has prompted the development of methodologies to generate cardiomyocytes (CMs), both *in vitro* and *in vivo* [3]. Human pluripotent stem cells (hPSCs), including embryonic stem cells (ESCs) and induced pluripotent stem cells (iPSCs), have nearly unlimited self-renewal capability and have the ability to differentiate into all cell types of the human body in principle [4]. These features make PSCs attractive as the source for large-scale production of CMs *in vitro*. Furthermore, patient-specific cardiomyocytes can not only be used in cell replacement therapy, it may also find applications in pharmaceutical drug testing and disease modeling for pathological studies.

With the advent of iPSCs generated a decade ago, transcription factors (TF) showed their powerful capability to direct cell fate [5–7]. Currently, virus-mediated transfection or called viral transduction, such as adenovirus, lentivirus, retrovirus and sendai virus, are widely used for forced expression of TFs in eukaryotic cells [8–12]. These DNA-based approaches, however, pose the potential for insertional-/recombination-mediated chromosomal disruptions and often result in incomplete silencing of exogenous gene expression, thus the cells derived by these methods have limited clinical applicability [13, 14]. Here, we employed a transient protein delivery tool, bacterial type III secretion system (T3SS), to translocate TFs in the form of protein directly inside mammalian cells. The T3SS is encoded by certain Gram-negative bacteria, forming a nanosyringe-like structure on the bacterial surfaces [15]. Bacterial pathogens utilize the T3SS to inject (translocate) virulence proteins into eukaryotic host cells, which facilitate the survival and reproduction of the bacterial cells inside the host environment [16]. All T3S-dependant effectors harbor a short N-terminal secretion signal which binds to chaperones that stabilize them inside the bacterial cells, facilitating their interaction with secretion machinery for secretion [17, 18]. This process of secretion is powered by the ATPase associated with the type III secretion machinery inside the bacterial cells [19].

*Pseudomonas aeruginosa* is a well-studied opportunistic human pathogen which injects proteinous exotoxins (ExoS, ExoT, ExoY, and ExoU) directly into host cells via the T3SS [20, 21]. Since this naturally occurring protein injection machinery does not involve bacteria entering the host cells, attenuated *P*. *aeruginosa* strain is ideal for the expression and delivery of exogenous TFs via its T3SS. By fusing with the T3SS secretion signal (N-terminal 54 amino acids) of ExoS, many functional proteins, such as nucleases, tumor-associated antigens and transcription factor, have been successfully delivered into various mammalian cells [22–27]. Recently, a study of human cardiomyocyte reprogramming has demonstrated that five transcriptional factors, GATA4, MEF2C, TBX5, ESRRG and MESP1 (abbreviated as GMTEM), were required for cardiac gene-expression and phenotypic shifts from human fibroblasts [28]. However, fibroblasts are not a proper source for large-scale production of cardiomyocytes owing to theirs limit proliferation capacity when cultured *in vitro*. In this study, the GMTEM transcription factor cocktail was delivered inside hPSC-derived cells via the bacterial T3SS and a significant enhancement of the *de novo* cardiomyocytes differentiation from human PSCs has been observed, paving the ground for the future application of the T3SS mediated TF delivery technology for various biomedical applications.

## Materials and methods

### Bacterial strains and plasmids

The bacterial strains and plasmids used in the present study are listed in Table 1. All *P*. *aeruginosa* strains were grown in Luria broth (LB) or on L-agar (LA) plates at 37°C. Carbenicillin was used at a final concentration of 150 μg per mL to maintain selection for the plasmid.

**Table 1:** Plasmids and strains used in this study.

Plasmid and strain	Description	Source
Plasmids		
pExoS_54_F	Promoter and N-terminal 54 aa of ExoS fused with FLAG tag in *Escherichia–Pseudomonas* shuttle vector pUCP19; Cb^r^	[42]
pExoS_54_F-GATA4	pExoS_54_F fused with *gata4* gene; Cb^r^	This study
pExoS_54_F-MEF2c	pExoS_54_F fused with *mef2c* gene; Cb^r^	This study
pExoS_54_F-TBX5	pExoS_54_F fused with *tbx5* gene; Cb^r^	This study
pExoS_54_F-ESRRG	pExoS_54_F fused with *esrrg* gene; Cb^r^	This study
pExoS_54_F-MESP1	pExoS_54_F fused with *mesp1* gene; Cb^r^	This study
*P*. *aeruginosa*		
PAK-J	Wild-type *Pseudomonas aeruginosa* strain with enhanced T3SS	[22]
Δ8	PAK-J deleted of *exoS*, *exoT*, *exoY*, *ndk*, *xcpQ*, *lasR-I*, *rhlR-I* and *popN*	[43]
Δ*exsA*	PAK-J deleted of *exsA*	[25]

### Generation of transcription factors delivery constructs

An expression vector, pExoS_54_F, contains the promoter of T3SS effector ExoS and its N-terminal 54 amino acids of ExoS (ExoS_54_), followed by a Flag-tag in the multiple cloning site (MCS) of *E*. *coli-Pseudomonas* shuttle vector pUCP19 (Table 1). Five human transcriptional factor genes, *gata4*, *mef2c*, *tbx5*, *esrrg*, and *mesp1* were each cloned into the pExoS_54_F following PCR amplification and restriction enzyme digests, generating in-frame fusions behind the ExoS_54_-Flag fragment, resulting in pExoS_54_F-GATA4, pExoS_54_F-MEF2c, pExoS_54_F-TBX5, pExoS_54_F-ESRRG, and pExoS_54_F-MESP1, respectively.

### Cell culture and cardiomyocyte differentiation

A human ESC line (RUES2) and a human iPSC line (iPS-3) were grown on Vitronectin coated plates (Life Technologies, Carlsbad, CA) in Essential 8 medium (Life Technologies) and passaged after dissociation by 0.5 mM EDTA (Life Technologies). The hESC RUES2 was purchased from WiCell Research Institute (Madison, WI, http://www.wicell.org); and iPS-3 was generated from foreskin fibroblasts of a healthy individual (catalog no. CRL-2522; American Type Culture Collection, Manassas, VA, http://www.atcc.org) using Sendai virus SsVdp(KOSM)302L kindly provided by Dr. Mahito Nakanish in accordance with a published protocol [11]. The iPS-3 was generated under an approved University of Florida Environmental Health and Safety biosafety approval number RD-3933. All PSCs were cultured at 37°C with 5% CO_2_. Ciprofloxacin was used at a final concentration of 20 μg/mL to clear the *P*. *aeruginosa* cells. Human PSC-derived cardiomyocytes were generated following the protocol as described previously [29]. In brief, Essential 8 medium was replaced with RPMI-B27 medium (Invitrogen) supplemented with the following cytokines: 100 ng/mL human recombinant activin A (R&D Systems) for 24 h, followed by 10 ng/mL human recombinant BMP4 (R&D Systems) for 4 days. The medium was then changed for RPMI-B27 without supplementary cytokines; cultures were refed every 2 days for 2–3 additional weeks. Widespread spontaneous contraction was typically observed by day-14.

### Cytotoxicity assays

Human ESCs was co-cultured with *P*. *aeruginosa* strains for various period of time at indicated multiplicity of infection (MOI). After the co-culture, 50-μL cell culture supernatant per sample was used for lactate dehydrogenase (LDH) release assay by CytoTox96 (Promega) following the manufacturer’s instruction. For crystal violet staining, bacteria and floating PSCs were removed by washing three times in phosphate-buffered saline (PBS) and adhered cells were stained with 0.25% crystal violet.

### Protein translocation assay

Human ES cells were seeded at approximately 70–80% confluence in antibiotic-free medium. *P*. *aeruginosa* strains were grown at 37°C in Luria broth containing carbenicillin until reaching an optical density (OD_600_) of 0.8. hESCs were co-cultured with bacteria at an MOI of 30 or 150 for 3 hours. Co-culture was terminated by washing cells 3 times with PBS to remove the vast majority of bacterial cells. For Western Blot analysis, cells were collected by digestion with TrypLE (Gibco) and centrifuged at 200 × g for 5 min. For continuing the culture, cells were grown on Essential 8 medium containing 20 μg per mL ciprofloxacin to eliminate the residual *P*. *aeruginosa* cells.

### Western blot assay

For Western blot analysis of the injected proteins, the cells were collected right after bacterial infection (see above). The cell pellets were lysed with 40 μL PBS containing 0.2% Triton-X (bacterial cells do not get lysed) on ice for 10 minutes. The nuclear protein extracts were prepared using an extraction kit from Beyotime Biotechnology (Shanghai, China) and followed the manufacturer’s instruction. The soluble fraction was collected, mixed with an equal volume of 2 × sodium dodecyl sulfate-polyacrylamide gel electrophoresis (SDS-PAGE) loading buffer and boiled for 15 minutes. Protein samples were separated on 4–20% gradient SDS-PAGE gels (Bio-Rad), transferred onto polyvinylidene fluoride (PVDF) membrane, and then probed with anti-Flag antibody (mouse M2 monoclonal Ab; Sigma) for translocated fusion proteins and anti-β-Actin (Santa Cruz) for internal control.

### Immunocytochemistry

For ExoS_54_-Flag-TFs fusion staining, the hESCs were co-incubated with indicated bacterial strains for 3 h. The infection was stopped by washing cells with PBS, then the hES cells were treated with 20 μg per mL ciprofloxacin for another 1–2 h, then fixed for immunostaining. For cardiomyocyte-specific protein staining, the hESC-CMs on differentiation day-14 were used to conduct the *in situ* staining. Cells were fixed with 4% paraformaldehyde in PBS for 20 min at room temperature, then permeabilized with 0.1–0.2% Triton X-100 in PBS and blocked with 10% goat serum in PBST (1 × PBS with 0.05% Triton X-100) for 1 h. Cells were incubated with primary anti-Flag monoclonal antibody (Sigma) diluted 1:1000 or anti-α-actinin (Sarcomeric) monoclonal antibody (Sigma) diluted 1:800, for 2 hours at room temperature, then washed 3 times in PBST. Cells were then incubated with secondary antibody (Alexa Flour 488-conjugated anti-mouse IgG, 1:200) for 1 hour at room temperature, washed with PBST for 3–4 times, then mounted and stained nucleus with DAPI and examined under fluorescence microscope.

### Flow cytometry

Single cells of hPSC-CM culture were dissociated on day-14 using TrypLE (Gibco) and fixed with 4% paraformaldehyde (Sigma-Aldrich) for 20 min at room temperature. Cells were permeabilized in 0.1% saponin/PSB solution and stained with anti-Flag antibody (Sigma), or anti-troponin T cardiac isoform monoclonal antibody (Thermofisher) labeled with Alexa-488 using Zenon technology (Molecular Probes/Invitrogen). Stained cells were analyzed on a FACS Calibur flow cytometer (BD-Biosciences).

### Quantitative real-time PCR

Total RNA was isolated from PSCs-derived cells collected on day-14 of differentiation protocol with the use of the RNeasy mini kit (Qiagen). DNAse I (Turbo DNA-free, Ambion) was used to eliminate contaminating genomic DNA. The first-strand cDNA was synthesized with High Capacity cDNA Reverse Transcriptase Kit (Applied Biosystems). The Power SYBR Green PCR Master Mix (Applied Biosystems) was used to perform real-time PCR reaction, according to the manufacturer’s instructions. Data were analyzed using the 2^−ΔΔCt^ method [30], using a stable reference gene (*gapdh*) as the normalization control. The fold changes in mRNA expression were relative to the hPSC-derived cells (day-14) without bacterial infection.

### Statistical analysis

Statistical analysis was carried out using GraphPad Prism software. Data were presented as the mean of the biological replicates and error bars represented standard deviations. Student’s *t*-test (two-tailed) was performed to assess the statistical significance of the data analysis.

## Results

### Cytotoxicity of attenuated *P*. *aeruginosa* strain

**Fig 1:**
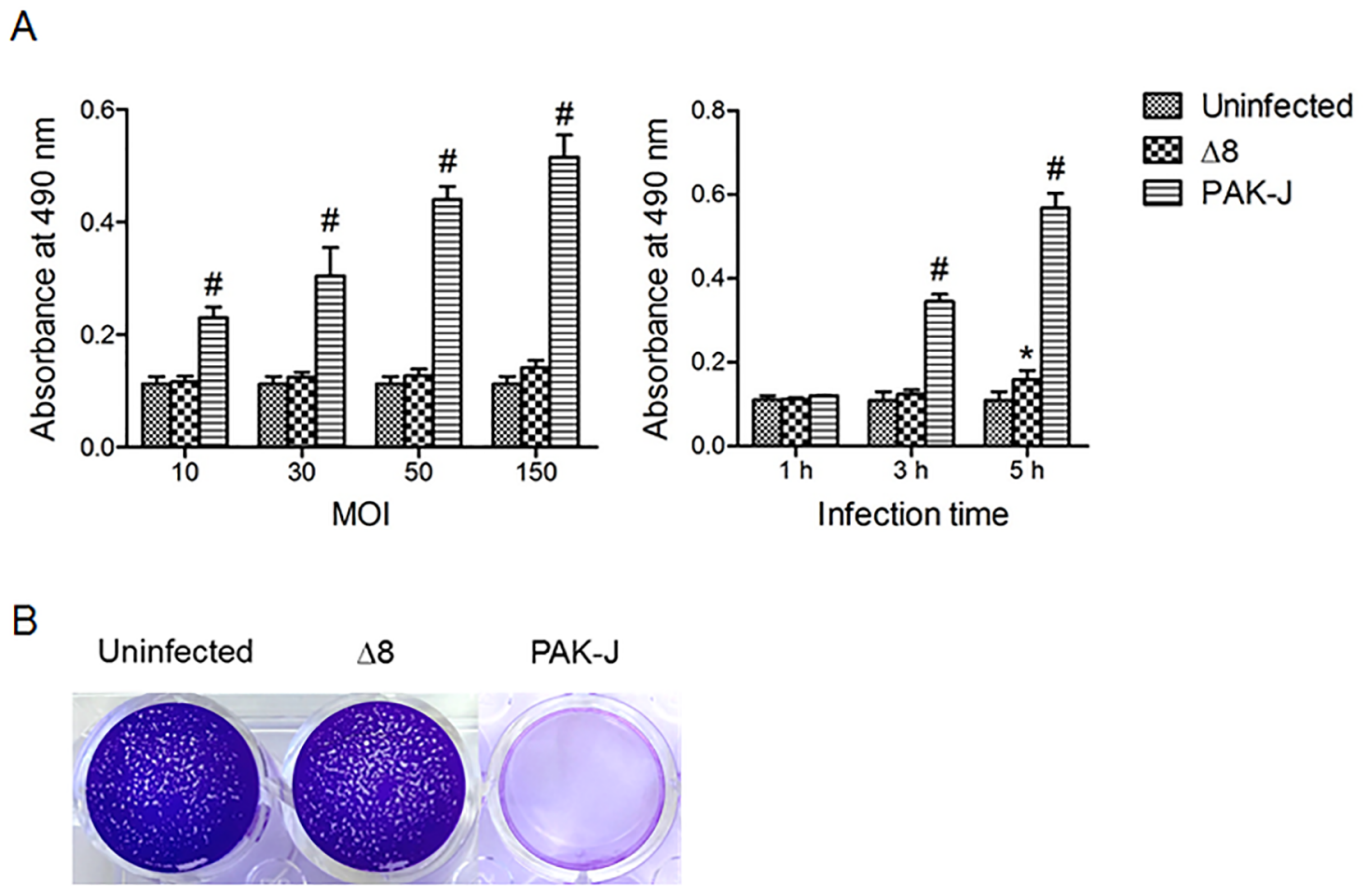
Cytotoxicity assay of *P*. *aeruginosa* strain Δ8. (A) Human ES cell line RUES2 was co-cultured with strain Δ8 or wild-type strain PAK-J for 3 h at indicated MOI (left panel), or for the indicated length of time at MOI of 50 (right panel). After co-culture, the culture supernatants were used for lactate dehydrogenase (LDH) release assay. Data represent means of three biological replicates. Error bars represent SD. *P < 0.05, ^#^ *P* < 0.001. (B) Crystal violet staining of the viable hES cells that remain adhered to tissue culture plates after 3 h co-culture with indicated bacterial cells at MOI of 150.

To use *P*. *aeruginosa* as a protein delivery vehicle on hPSCs, the bacterial virulence needs to be minimized. Total 8 genes implicated in the bacterial cytotoxicity were deleted from the *P*. *aeruginosa* PAK-J, resulting in an attenuated strain Δ8 (Table 1). To assess the cytotoxicity of this strain on human PSCs, human ES cell line RUES2 was infected by the Δ8 at various MOI for different length of time and the lactate dehydrogenase (LDH) released from hESCs was detected. As the results shown in Fig 1A, the bacterial cytotoxicity increased gradually along with the increase of MOI or co-culture time. Comparing with uninfected control, Δ8 showed no significant cytotoxicity within 3 h of co-culture, while the wild-type strain PAK-J showed significant cytotoxicity at MOI as low as 10 for 3 h. Furthermore, the viable hES cells that remain adhered to tissue culture plates after 3 h of co-culture at MOI of 150 were stained with crystal violet (Fig 1B). The hESCs remained adhered to the plate following co-incubation with strain Δ8, whereas co-incubation with the wild-type strain PAK-J resulted in a complete loss of the adhering cells.

### T3SS mediated delivery of transcription factors into hESCs

**Fig 2:**
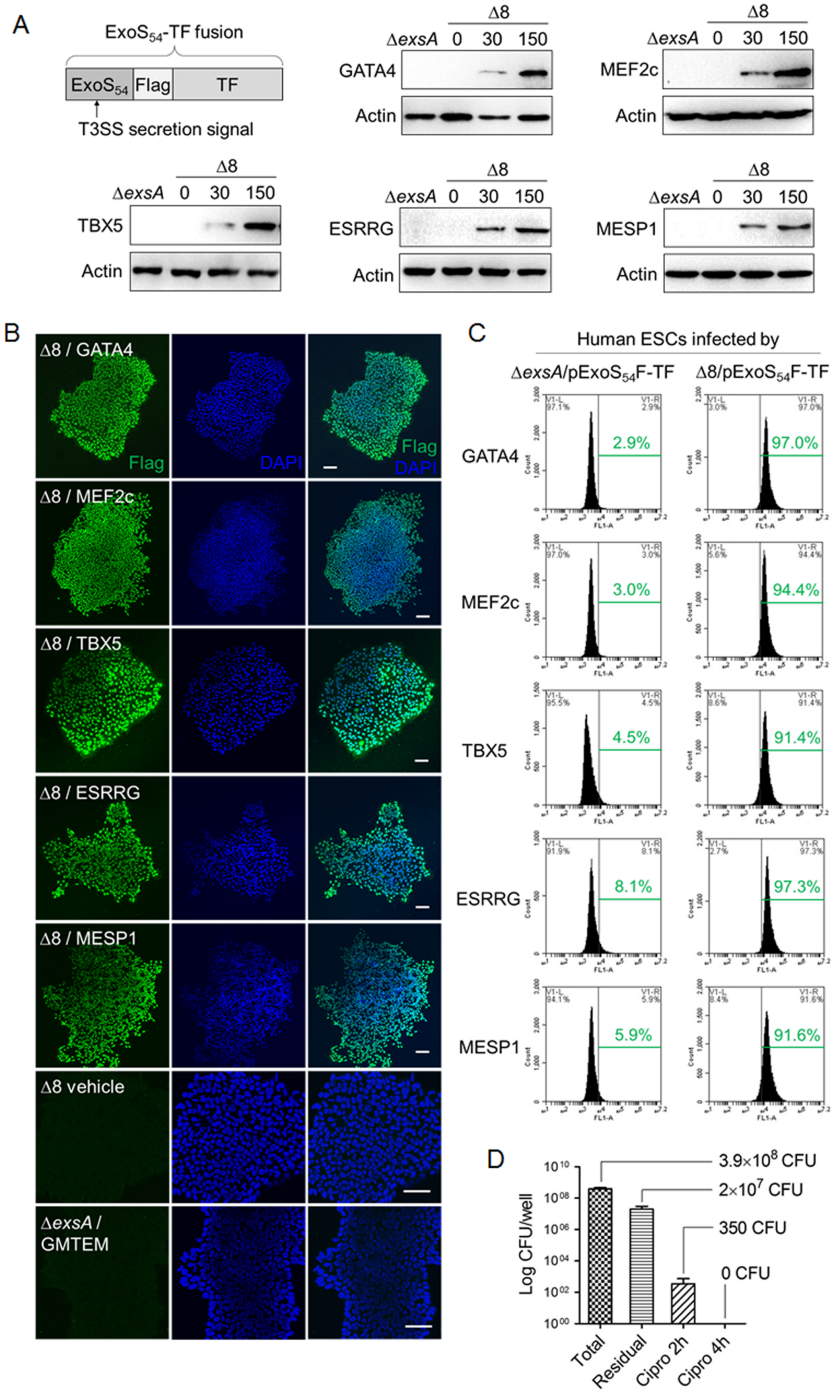
T3SS-based injection of transcription factors into hESC. (A) hESCs were co-cultured with strain Δ8 containing indicated ExoS_54_-TF fusions at indicated MOI (0, 30 or 150) for 3 hours. Cells were lysed, nuclear protein extracted and examined for injected proteins by anti-Flag immunoblot. The T3SS deficient strain Δ*exsA* was infected at MOI of 150. (B) hESCs were co-cultured with indicated strains for 3 hours at MOI 50 and immunostained with anti-Flag to illuminate translocated ExoS_54_-Flag-TF proteins. The hESCs were infected with Δ8 (MOI = 150) or Δ*exsA* containing each of the ExoS_54_-TF fusion at MOI of 30 per strain (total MOI of 150, Δ*exsA/*GMTEM) as negative controls. Nuclei were stained with DAPI. Scale bar = 100 μm. (C) Translocation efficiency of ExoS_54_-Flag-TF fusions into hESCs was analyzed by FACS. (D) hESCs was co-cultured with Δ8 at MOI of 150 for 3 hours. Supernatants and adherent ES cells of each well were collected and serially diluted, then plated on LB-agar plates to enumerate the total bacterial cell number (CFU/well) and bacteria attached to the hES cells (residual), respectively. Co-culture was terminated by washing cells with PBS and continued to grow the hES cells on culture medium containing 20 μg/mL ciprofloxacin. After antibiotic treatment for 2 and 4 h, hES cell colonies were scraped and lysed with 0.2% Triton-X100 (bacterial cells do not get lysed), the lysates were serially diluted and plated on LB-agar plates to calculate the residual bacterial cell numbers (CFU/well). Error bars represent SD of triplicate assays.

Experimental data from the above studies demonstrated that the engineered *P*. *aeruginosa* Δ8 strain has no detectable cytotoxicity to hESCs within 3 hours of co-culture at MOI≤150. Next, we tested if the delivery of transcription factors can be achieved within 3 hours by using the Δ8 strain. Genes encoding transcriptional factors GATA4, MEF2c, TBX5, ESRRG and MESP1 were each cloned into an expression vector pExoS_54_F, generating in-frame fusions behind the T3SS secretion signal ExoS_54_ and with a Flag-tag in the middle (Fig 2A). The resulting expression vectors pExoS_54_F-GATA4, pExoS_54_F-MEF2c, pExoS_54_F-TBX5, pExoS_54_F-ESRRG and pExoS_54_F-MESP1 (Table 1) were each introduced into the Δ8 strain as well as a T3SS deficient strain Δ*exsA* by electroporation. The resulting strains were then individually co-cultured with hESCs at an MOI of 30 or 150 for 3 hours. Free-floating bacterial cells were subsequently removed by successive washes with PBS, and the hESCs were examined for intracellular fusion proteins by both Western blot and immunohistochemistry using anti-Flag antibody. As the results shown in Fig 2A and 2B, all of the TF fusion proteins could be translocated into hESCs by the Δ8 strain in a dose-dependent manner, as higher level of intracellular fusion proteins were observed when the MOI increased from 30 to 150; while T3SS deficient strain Δ*exsA* failed to deliver the TFs into the hESCs, indicating that the TF delivery was T3SS dependent and the delivery amounts can be fine-tuned by MOI. The fluorescence-assisted cell sorting (FACS) assay of hESCs showed that the efficiency of T3SS-mediated TFs delivery was > 90% at an MOI of 30 for 3 hours (Fig 2C). The above results demonstrate that *P*. *aeruginosa* strain Δ8 is competent for high-efficiency TFs delivery into hESCs with negligible cytotoxicity.

Following 3 h of co-culture with Δ8 at MOI 150, the majority of the bacterial cells (~95%) can easily be removed by a washing step, leaving about 5% of the residual bacterial cells remain attached to the ES cells (Fig 2D). To eliminate the residual bacterial cells thoroughly, the hES cells were sub-cultured in medium containing ciprofloxacin which is a potent antibiotic against *P*. *aeruginosa*. hESCs were scraped off from the plate at various time points and viable bacterial cells were enumerated by plating on L-agar medium. The number of viable bacterial cells rapidly decreased in the ciprofloxacin-treated hESC samples, with no detectable bacterial cells by 4 hours of the antibiotic treatment (Fig 2D).

### Optimal time point for TFs delivery during cardiomyocyte differentiation

**Fig 3:**
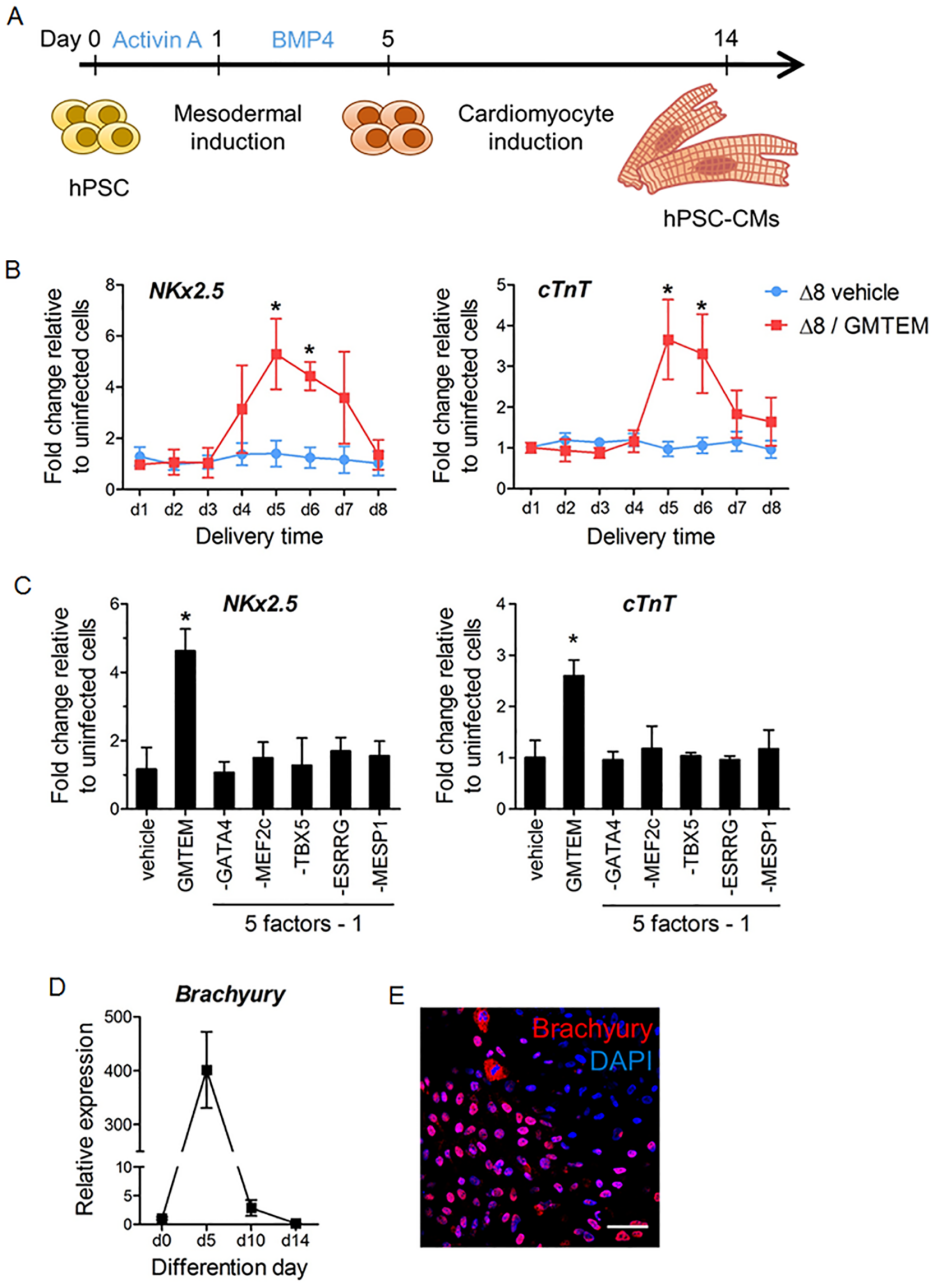
Optimal delivery time of the GMTEM during cardiomyocytes *de novo* differentiation. (A) Protocol for *in vitro* differentiation of cardiomyocytes from human pluripotent stem cells (hPSCs). (B) Differentiating ESCs were infected with the Δ8 (vehicle, MOI = 150) or Δ8/GMTEM (MOI of 30 per strain) on the indicated days, respectively. Total RNAs were extracted on day-14. Expression levels of *nkx2*.*5* and *cTnT* were measured by qRT-PCR. Data represents as fold change expression relative to the uninfected control on day-14. *P < 0.05 (n = 3). (C) Various TF combination delivered on day-5, qRT-PCR measurements of *nkx2*.*5* and *cTnT* expression in hESC-CMs on day-14. (D) Brachyury expression pattern during hESC-CMs differentiation determined by qRT-PCR. (E) Immunocytochemistry of hESC derived cells (on day-5) expressing mesodermal marker brachyury. The bar is 100 μm.

To examine whether GMTEM can exert their cardiomyogenic effect in *de novo* human cardiomyocytes differentiation, we delivered GMTEM to a feeder-free hESC monolayer for differentiation, following a protocol described by Laflamme *et al*. [29]. Briefly, a 70–80% confluence human ESC culture was first treated with a high dose of activin A in a BPMI-B27 medium for 24 h, followed by 4 days of treatment with bone morphogenetic protein 4 (BMP4) to induce mesodermal cells. Then, the cells were cultured in BPMI-B27 medium without any supplementary factor and contracting cardiomyocytes are typically observable by day-14 (Fig 3A). This robust protocol resulted in consistent cardiomyocyte differentiation efficiency (20–30%) that is low enough to address the contribution of GMTEM to the differentiation process.

The GMTEM five factors were delivered once with MOI = 30 per strain (total MOI of 150) from day-1 to day-8 during the above cardiomyocyte differentiation process. On day-14, total RNA of the hESC-CMs was extracted and the expression levels of cardiac marker genes *Nkx2*.*5* and *cTnT* were determined by quantitative real-time PCR (qRT-PCR). As shown in Fig 3B, comparing to the cells infected by strain Δ8 without any expression vector (vehicle), GMTEM delivery on the first 3 days of differentiation showed no promoting effect on the *Nkx2*.*5* and *cTnT* gene expression; GMTEM injection on day 5 to 6 resulted in the most significant increase of both *Nkx2*.*5* and *cTnT* expression levels; nevertheless, after this time window, GMTEM showed incompetent in upregulating the expression of *Nkx2*.*5* and *cTnT*. Accordingly, day-5 was chosen as the optimal GMTEM delivery time point for the most effective promotion of cardiomyocyte-like cells differentiation. At this day, all of the five factors were required for the enhanced cardiac differentiation, withdraw of any one factor resulted in a loss of the promotion (Fig 3C). The expression of mesodermal marker *brachyury* during hESCs-CMs differentiation was also determined by qRT-PCR and immunocytochemistry. As shown in Fig 3D, hESCs derived cells showed the highest *brachyury* expression level on day-5 of the differentiation protocol. Indeed, most hESC derived cells expressed detectable Brachyury in the nucleus on day-5 (Fig 3E). These results demonstrated that GMTEM exert their effects most optimally on mesodermal cells for cardiac specification.

### GMTEM promote hESCs differentiation into cardiomyocytes

**Fig 4:**
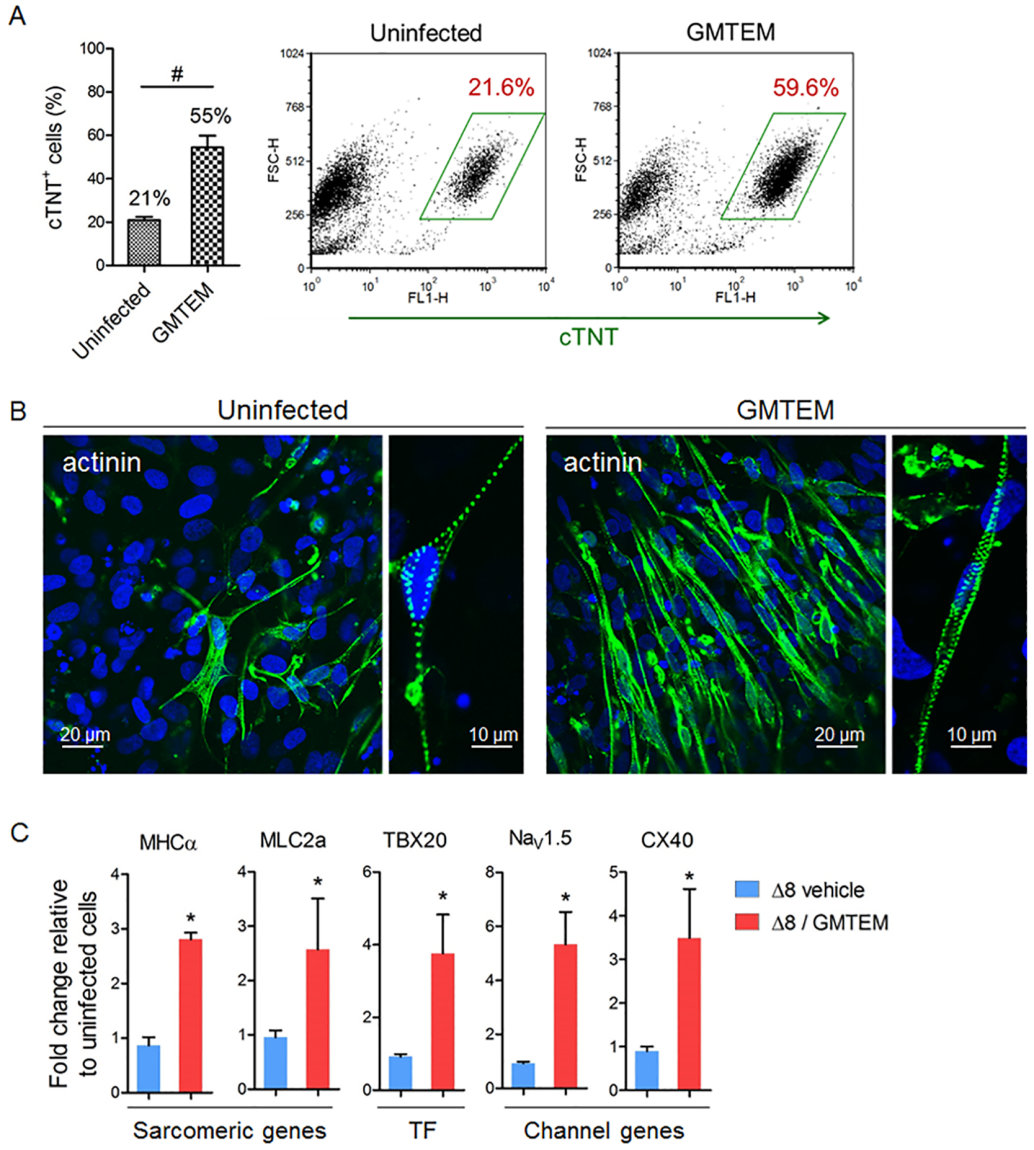
GMTEM delivery enhances hESC-derived CMs differentiation. (A) FACS analysis and the percentage of ESC derived cTnT^+^ cardiomyocytes. Bar figure show average of three repeats. ^#^ *P* < 0.001. (B) Detection of cardiomyocyte-specific protein sarcomeric α-actinin in the hESC-CMs. (C) Relative expression levels of cardiac marker genes in the hESC-CMs. *P < 0.05 (n = 3).

We delivered GMTEM together into hESC derived mesodermal cells by bacterial T3SS. After 14 days of differentiation, the hESC-CMs displayed spontaneous rhythmic contractile movements (S1 video). The cTNT positive cell population was determined by FACS, average 21% of the cTnT^+^ cells appeared in the uninfected control, while GMTEM delivery on day-5 resulted in average 55% of cTnT^+^ cells (Fig 4A). We also analyzed the cardiac marker protein expression of sarcomeric α-actinin using immunocytochemistry and observed that the proportion of actinin positive cells was obviously higher in the GMTEM induced culture than that of uninfected control, and the hESC-CMs were revealed of well-organized cross-striations (Fig 4B). Using qRT-PCR analysis, we evaluate the effect of exogenous GMTEM delivery on the expression of selected cardiac genes in hESC-CMs (Fig 4C). The gene expression of the sarcomeric proteins MHCα (*MYH6*) and MLC2a (*MYL7*), the transcription factor TBX20, the ion channel proteins Nav1.5 (*SCN5A*) and CX40 (*GJA5*) was significantly higher in the GMTEM delivered group. These results demonstrated that transient GMTEM proteins delivery induced cardiac gene-expression and enhanced the hESC-CMs differentiation.

### GMTEM promote hiPSCs differentiation into cardiomyocytes

**Fig 5:**
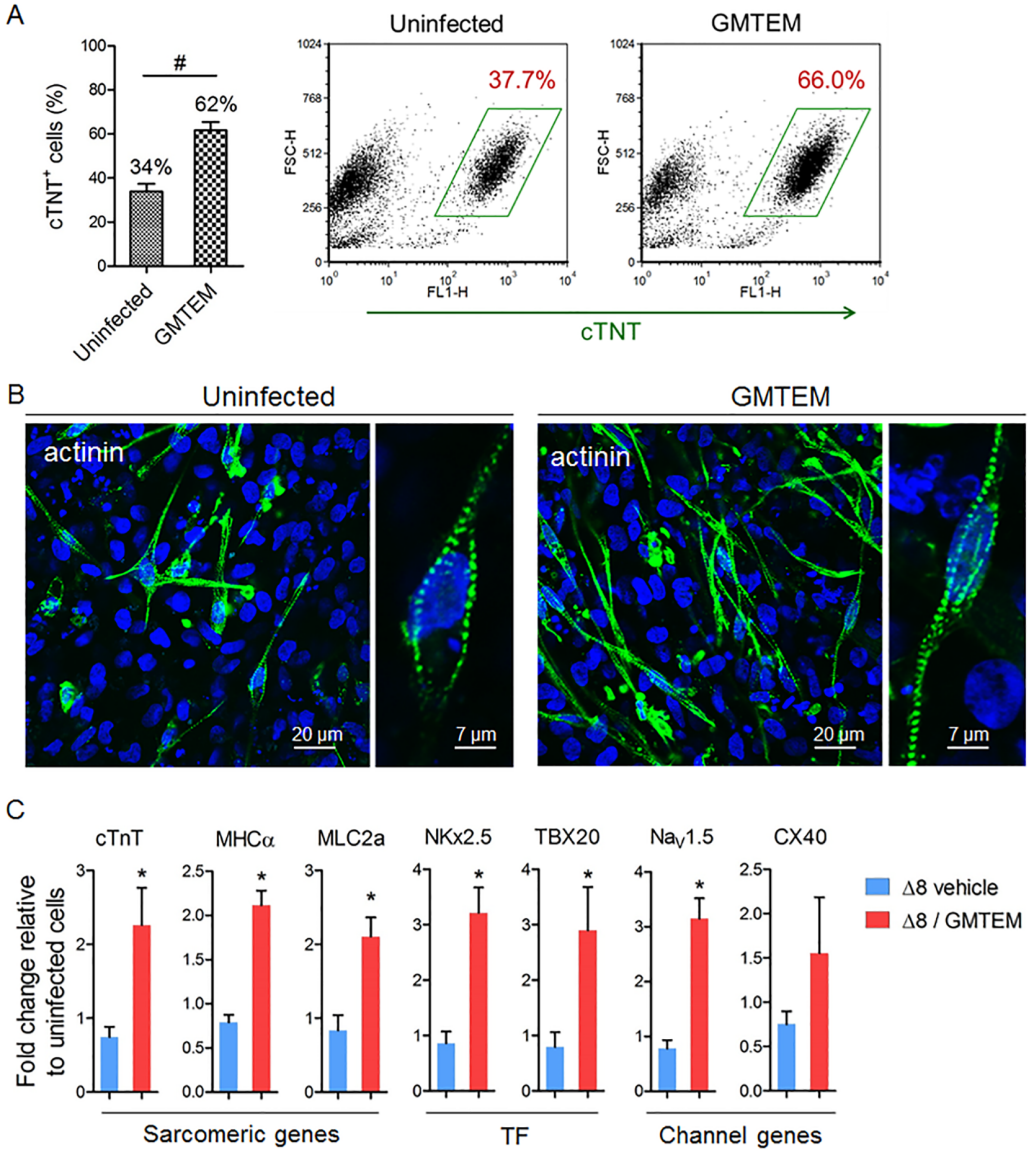
GMTEM delivery enhances hiPSC-derived CMs differentiation. (A) FACS analysis and the percentage of human iPSC derived cTnT^+^ cardiomyocytes. Bar figure show average of three repeats. ^#^ *P* < 0.001. (B) Detection of sarcomeric α-actinin expression in the hiPSC-CMs. (C) Relative expression levels of cardiac marker genes in the hiPSC-CMs. *P < 0.05 (n = 3).

The breakthroughs in the field of iPS cell technology enlisted human iPSCs as an additional source for *in vitro* differentiated cardiomyocytes, sharing similarities with their hESC-derived counterparts [31, 32]. Moreover, patient-specific iPSCs can faithfully recapitulate disease phenotypes *in vitro* when differentiated into disease-relevant cell types, opening the doors for its use in disease modeling and drug discovery [33]. Here, we asked if a combined delivery of the GMTEM could also enhance the efficiency of human iPSC-CMs differentiation. Using the same differentiation procedure as described above for the hESC-CMs, GMTEM were delivered on day-5 using bacterial T3SS. After 14 days of differentiation, the hiPSC-CMs culture displayed widespread spontaneous contraction (S2 video). Average 34% of the cTnT^+^ cells appeared in the uninfected control, while GMTEM delivery resulted in average 62% of cTnT^+^ cells (Fig 5A). Immunocytochemistry demonstrated that the proportion of sarcomeric α-actinin positive cells was higher in the GMTEM induced cultures, and the hiPSC-CMs were also revealed of well-organized cross-striations (Fig 5B). Meanwhile, GMTEM delivery leads to significant increase in the expression of cardiac-specific markers cTnT, MHCα, MLC2a, NKx2.5, TBX20, Nav1.5 and CX40 (Fig 5C). All of these data clearly indicated that GMTEM cocktail effectively enhances the iPSC-CMs differentiation.

## Discussion

**Fig 6:**
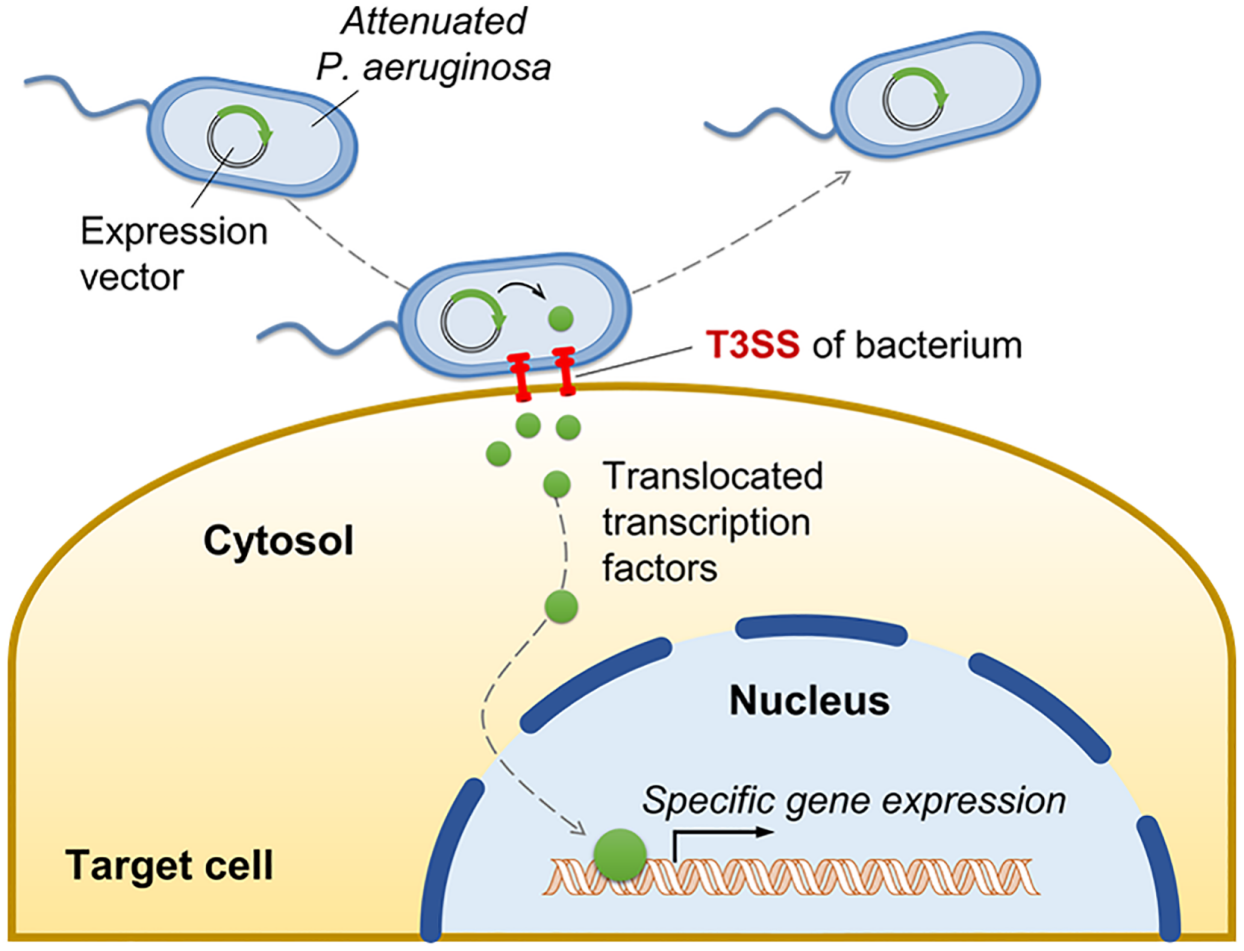
Schematic representation of T3SS-based transcription factor (TF) delivery.

In this study, we demonstrated that a bacterial type III secretion system (T3SS)-mediated protein delivery method could be utilized to deliver TFs into human PSCs to promote cardiomyocytes differentiation. By expressing in-frame fusions of T3SS secretion signal ExoS_54_ and TF genes inside *P*. *aeruginosa* cells, TF proteins could effectively be translocated into host cell cytosol when bacteria make contact with PSCs, a condition known to activate T3SS. The translocated TFs are targeted to nucleus with their own nuclear localization signals [34] and exert their biological functions (Fig 6). Using this protein delivery method, TFs could efficiently be injected into the PSCs directly, avoiding the introduction of foreign genetic materials. Also, due to the simplicity of manipulation and limited intracellular half-lives of the injected proteins, TFs can be delivered for a desired period of time with variable doses, therefore, it is suitable for large-scale preparation of cardiomyocytes.

Using the T3SS based protein injection tool, five transcriptional factors relevant to heart development, GATA4, MEF2C, TBX5, ESRRG, and MESP1, were injected into hPSC derived cells and observed an enhanced cardiomyocytes differentiation. Interestingly, our data showed that GMTEM delivery on a narrow time window of day-5 to 6 can significantly upregulate the cardiac marker genes expression and improve the ultimate differentiation of hPSC-CMs. As the expression of mesodermal marker gene *brachyury* peaked at day-5, our results indicated that GMTEM delivery right after the mesodermal induction stage enhanced the cardiomyocyte differentiation, while earlier or later delivery diminished the enhancing effect. One explanation of this time-dependent event might be that the dynamic chromatin remodeling on specific loci plays an important role during pluripotent cells differentiation. Mammalian DNA is highly compacted into chromatin which must be locally remodeled to permit transcription [35]. At its essence, cell type-specific transcription is achieved through the selective remodeling of chromatin at discrete loci to allow the productive engagement of polymerase II. During the PSC differentiation in this study, the GMTEM’s target loci are likely transiently remodeled to permit transcription and permissive for a cardiac lineage-specific commitment on day-5 to 6, while they are inaccessible outside of this narrow time window.

The recent application of genome-wide techniques to human ESCs has started to shed light on the epigenetic dynamics that regulate ‘‘localized” chromatin remodeling and accessibility to transcription factors in different cell types, including cardiomyocytes [36, 37]. Of note, a cardiac-specific subunit of BAF chromatin-remodeling complexes, BAF60c (also called SMARCD3), was described to directly interact with cardiac transcriptional activators [38] and involved in regulation of mesodermal lineage commitment [39]. The cardiogenic role of BAF60c depends on an interaction with cardiac transcription factor GATA4 and a supporting role of Tbx5 [40]. Together, these factors promote differentiation of early mouse mesoderm into heart [41], suggesting the ability to direct uncommitted mesoderm to the cardiac fate. In the future, T3SS-based protein delivery system could be used to deliver BAF60c together with the minimal set of TFs during PSC differentiation to enhance the cardiomyocytes differentiation.

## Conclusions

Here, we have utilized bacterial type III secretion system (T3SS) to deliver transcription factors in the form of protein directly into human PSCs. The delivery efficiencies of transcription factors were > 90% in a short infection time (3 hours). Simple antibiotic treatment could completely eliminate the residual bacterial delivery strain. Delivery of five transcriptional factor cocktail (GATA4, MEF2c, TBX5, ESRRG, and MESP1) relevant to early heart development was shown to promote both the human ESC and iPSC differentiation into functional cardiomyocytes. Of interest, a specific narrow window of time (day-5 to 6 of differentiation protocol) is required for the maximal effect of the delivered transcriptional factors. We expect that this efficient and transgene-free transcription factor delivery approach is applicable to a variety of other cell differentiation and reprogramming procedures.

## Supporting information

S1 Video The video of the beating area of the hESC-derived cardiomyocytes on the 14-day of differentiation (GMTEM delivered on day-5). (MP4)

S2 Video The video of the beating area of the hiPSC-derived cardiomyocytes on the 14-day of differentiation (GMTEM delivered on day-5). (MP4)
